# Differentiation mechanisms of rhizome clonal propagation strategies in *Leymus chinensis* based on physiological and metabolomic analyses

**DOI:** 10.3389/fpls.2026.1753652

**Published:** 2026-03-10

**Authors:** Yahui Zhang, Shimeng Zhao, Xiangyang Hou

**Affiliations:** 1 College of Grassland Science, Shanxi Agricultural University, Jinzhong, China; 2 Key Laboratory for Model Innovation in Forage Production Efficiency, Ministry of Agriculture and Rural Affairs, Jinzhong, China

**Keywords:** belowground–aboveground allocation, clonal plant ecology, physiological–metabolic regulation, rhizome-mediated clonal growth, source–sink relationship

## Abstract

*Leymus chinensis* is one of the dominant species in the Eurasian steppe, and its rhizome clonal propagation capacity is a key determinant of population propagation. However, the mechanisms underlying rhizome proliferation and differentiation among different *Leymus chinensis* germplasms remain unclear. In this study, two germplasms exhibiting markedly distinct rhizome clonal propagation capacities (E, Lc19; P, Lc13) were selected as experimental materials. Using non-destructive approaches under common-garden conditions, and by integrating physiological measurements with metabolomic profiling, we elucidated the mechanisms driving the differentiation in their clonal growth strategies. The results revealed that germplasm E (Lc19) adopts a “belowground investment” strategy, characterized by an optimized “small-leaf, long-culm” configuration, high photosynthetic and water-use efficiency, and preferential allocation of resources to rhizome internodes. Its rhizome internodes and nodes displayed a metabolomic profile with pronounced “growth engine” features, in which key primary metabolites, including several amino acids involved in protein synthesis and carbon–nitrogen metabolism, lipid-related compounds associated with membrane construction, and representative secondary metabolites such as flavonoids and phenolic acid derivatives (e.g., flavones and hydroxycinnamic acid–related compounds), were significantly enriched, supporting cell proliferation, tissue elongation, and oxidative homeostasis, thereby facilitating rapid rhizome propagation. In contrast, germplasm P (Lc13) followed an “aboveground maintenance” strategy, whereby its larger leaf area and higher transpiration rate enhanced aboveground resource competition. Its rhizome internodes and nodes exhibited a metabolic network dominated by homeostasis maintenance, with significant enrichment of metabolites related to energy metabolism and stress protection, including carbohydrates involved in energy supply and transport, as well as antioxidative compounds associated with redox regulation, forming a “homeostasis-centered defensive structure” that prioritized functional stability. This study clarified the intrinsic mechanisms underlying the intraspecific differentiation of rhizome propagation capacity in *Leymus chinensis* from a physiological–metabolic perspective, uncovered the metabolic basis of different ecological adaptation strategies, and provided theoretical and practical implications for the differentiated utilization of germplasms with distinct proliferation strategies in grassland restoration, stability maintenance, and ecological management.

## Graphical Abstract

**Figure:**
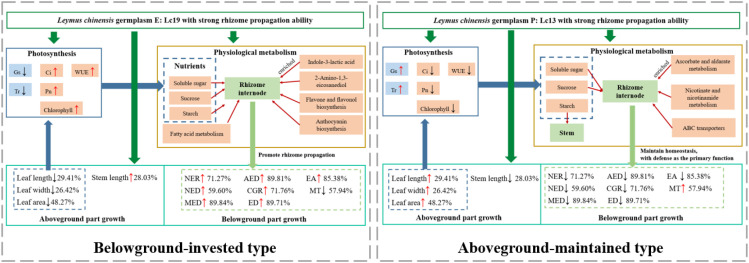

## 1 Introduction

Grasslands are an essential component of terrestrial ecosystems, playing an irreplaceable role in maintaining biodiversity and supporting livestock production (Yang and Peng, 2025). However, under multiple pressures such as global climate change and overgrazing, grassland degradation has become a severe global challenge. Currently, approximately 20%–35% of the world’s grasslands have experienced varying degrees of degradation (Han et al., 2020), and about 90% of natural grasslands in China are affected by degradation (Wang and Liang, 2023), posing a serious threat to ecological security and regional sustainable development. Against this background, identifying scientific vegetation restoration strategies to rehabilitate degraded grasslands has become a central issue in grassland ecology. During vegetation restoration, compared with seedlings derived from seed germination, clonal ramets originating from rhizome buds exhibit higher success rates in resource acquisition and stress tolerance due to their physiological integration with the parental plant (Lakoba et al., 2021; Kakusu et al., 2025). Therefore, rhizomatous clonal plants show great potential in stabilizing community structure and promoting ecological restoration (Evans et al., 2023). Their well-developed belowground rhizome systems serve not only as organs of clonal propagation but also as hubs for resource storage and transport, enabling plants to maintain population stability and propagation under stresses such as drought and nutrient deficiency through the integration and redistribution of photosynthates, water, and nutrients (Demetrio et al., 2024; Lubbe et al., 2023). Importantly, rhizomatous species do not rely on a single clonal propagation strategy. Instead, different species or genotypes may adopt contrasting reproductive and resource-allocation strategies, which are typically reflected in coordinated changes in physiological traits (e.g., photosynthetic performance, water-use efficiency, and carbon allocation patterns) and in the accumulation of specific groups of metabolites that support either rapid clonal expansion or functional stability (Pan et al., 2023; Liu et al., 2025; Morrison et al., 2024). These physiological and metabolic adjustments form the mechanistic basis of divergent rhizome-mediated propagation strategies. Elucidating the regulatory mechanisms underlying rhizome growth, particularly the physiological and metabolic bases of different clonal propagation strategies, is thus of great theoretical and practical importance for understanding ecological adaptation strategies and guiding degraded grassland restoration.

The growth and development of rhizomes is a complex process influenced by genetic regulation, environmental cues, and internal metabolic networks. Previous studies have identified 14 key transcription factors regulating rhizome growth in *Atractylodes macrocephala* (Ruan et al., 2021); nitrogen nutrition has been shown to be an important factor affecting axillary bud growth in the rhizomes of *Oryza longistaminata* (Shibasaki et al., 2021); and in *Panax ginseng*, research has revealed the central role of plant hormones in regulating rhizome bud dormancy and activation (Liu et al., 2023). However, these studies have largely focused on crops, medicinal plants, or model species, while research on key clonal species in natural grassland ecosystems remains lacking.

As a dominant and constructive species in the arid grasslands of eastern Eurasia, *Leymus chinensis* plays a crucial role in grassland ecosystems due to its strong clonal propagation capacity and ecological adaptability (Zhao et al., 2025). Its dense rhizome network effectively stabilizes soil, promotes nutrient cycling, and regulates the rhizosphere microenvironment through root exudates, thereby enhancing ecosystem stability and resilience (Lin et al., 2022). Notably, long-term natural selection and local adaptation have resulted in pronounced intraspecific differentiation among *Leymus chinensis* germplasms in rhizome propagation capacity. Some germplasms tend to prioritize belowground propagation through vigorous rhizome elongation and internode development, whereas others exhibit relatively conservative rhizome growth and allocate more resources to aboveground structures, reflecting contrasting clonal propagation and resource-allocation strategies (Bai and Hou, 2023). This differentiation provides ideal natural experimental materials for elucidating how contrasting clonal propagation and resource-allocation strategies are regulated at the physiological and metabolic levels within a single species.

However, current research on *Leymus chinensis* has primarily focused on physiological responses and gene functions, while the key regulatory bridge linking genotype to phenotype—the metabolic regulatory network—remains poorly understood. Systematic studies on the metabolic regulatory network underlying rhizome growth in *Leymus chinensis*, particularly those connecting key phenotypes with molecular mechanisms, are still lacking. Metabolites, as the final executors of biological processes and direct reflections of phenotypes, can most intuitively reveal the physiological state and regulatory endpoints of plants. Therefore, in this study, two *Leymus chinensis* germplasms (Lc19 and Lc13) with markedly different rhizome propagation capacities were used to integrate comprehensive phenotypic assessment, physiological analyses, and non-targeted metabolomics to: (1) characterize the physiological differences in growth, photosynthesis, and nutrient allocation between genotypes with different rhizome propagation capacities; (2) identify key differential metabolites and core metabolic pathways contributing to their phenotypic differentiation; and (3) construct a correlation framework linking “physiological traits–metabolic networks” to elucidate the metabolic basis of rhizome propagation in *Leymus chinensis*. This study deepens our understanding of ecological adaptation strategies in clonal plants and provides important theoretical foundations and metabolomic insights for breeding highly adaptive *Leymus chinensis* varieties and promoting grassland ecological restoration and sustainable management.

## 2 Materials and methods

### 2.1 Experimental materials

Based on the team’s previous experimental results, two *Leymus chinensis* germplasms exhibiting strong and weak rhizome clonal propagation capacities were selected as experimental materials, namely Lc19 and Lc13 (**Table 1**).

**Table 1:** Test material.

Rhizome propagative ability	*Leymus chinensis*	Longitudes	Latitude	Altitude/m	Location	Serial number
Excellent	Lc19	47°33′	124°14′	152	Fuyu County, Qiqihar City, Heilongjiang Province, China	E
Poor	Lc13	46°35′	121°26′	577	Horqin Right Front Banner, Hinggan League, Inner Mongolia Autonomous Region, China	P

### 2.2 Experimental design

**Figure 1:**
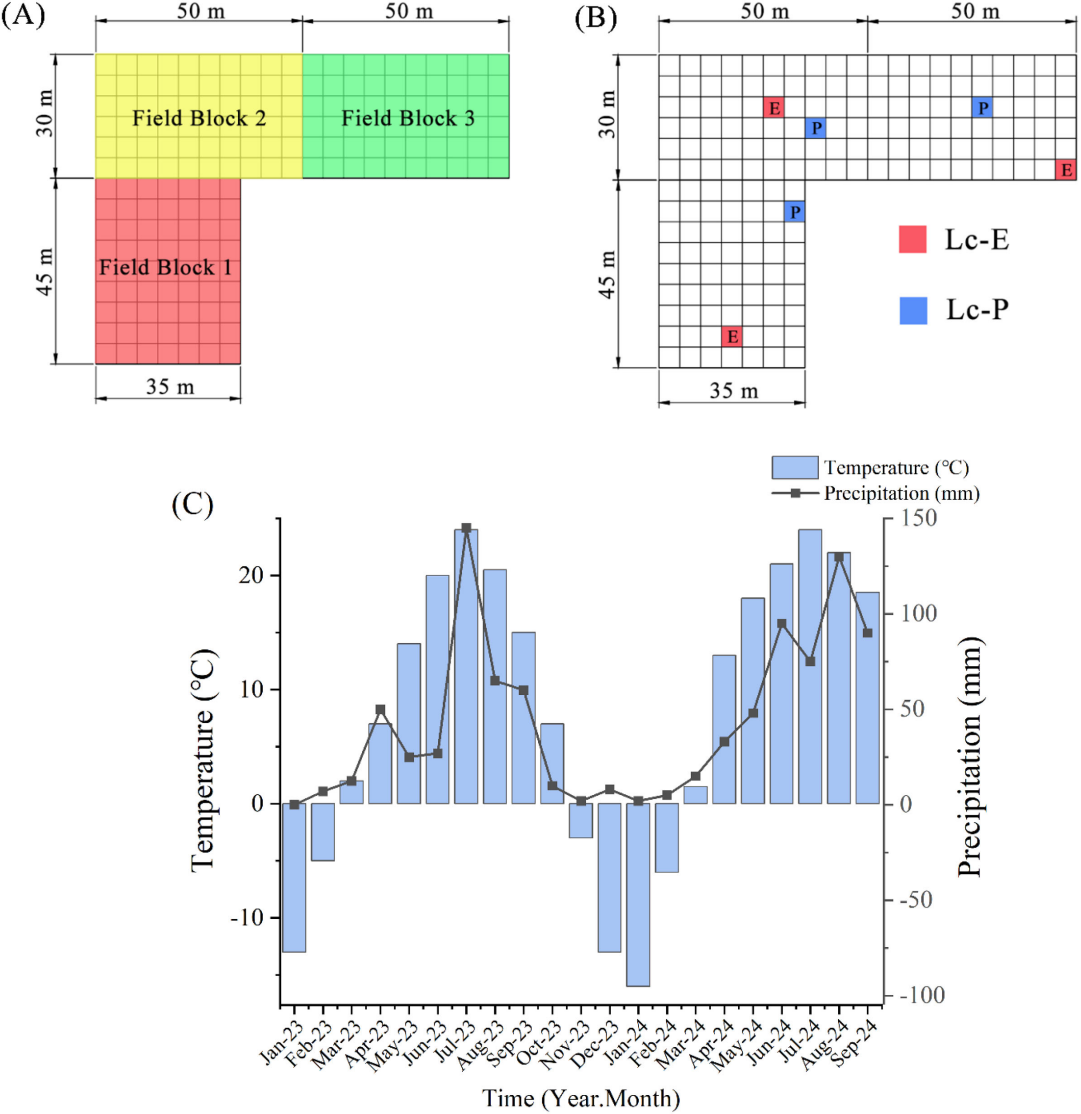
**(A)** Distribution of the experimental sites, **(B)** randomized planting layout of different *Leymus chinensis* germplasms, and **(C)** temperature and precipitation at the experimental site.

The experimental site is located at the *Leymus chinensis* rhizome plant experimental platform of Shanxi Agricultural University in Youyu County, Shuozhou City, Shanxi Province, China (112.29°E, 39.28°N; altitude of approximately 1400 m). The site is situated in a typical agro–pastoral ecotone of northern China and is characterized by a temperate continental monsoon climate. During the experimental period, the mean annual temperature was approximately 4.6 °C, and the mean annual precipitation was about 425 mm, with more than 60% of the rainfall occurring during July, August and September (**Figure 1C**). The frost-free period lasts approximately 100–120 days. The soil at the site is classified as light chestnut calcareous soil, which is representative of regional grassland habitats. The experimental field was divided into three blocks, each further subdivided into 5 m × 5 m plots to eliminate edge effects (**Figure 1A**). Two *Leymus chinensis* germplasms were arranged with three replicates and randomly planted within the three blocks (**Figure 1B**). In May 2023, *Leymus chinensis* plants were transplanted into the center of each plot, covered with soil and compacted, followed by initial flood irrigation to ensure successful establishment. After transplanting, no additional irrigation was applied, and plants were subsequently allowed to grow under local natural environmental conditions.

### 2.3 Measurement of phenotypic traits

In August 2024, during the early growth stage of *Leymus chinensis*, a randomized block sampling design was applied. Within each of the three experimental blocks, three uniformly growing individual plants were randomly selected for each germplasm, resulting in a total of nine biological replicates per germplasm. Plant height, stem length, leaf length, and leaf width were measured using a measuring tape.

### 2.4 Measurement of rhizome reproduction indices

Multiple of gents through tillering (MT): MT = number of tillers within the mother plant cluster/number of surviving transplanted mother plants.

Number of extravaginal ramets (NER): Number of ramets produced outside the mother plant cluster.

Clonal growth rate (CGR): CGR = number of rhizome-derived ramets/number of clonal growth days.

Number of rhizome extension directions (NED): The number of distinct directions in which rhizomes expanded.

Maximum one-direction extended distance (MED): The distance from the mother plant cluster to the farthest rhizome-derived ramet.

Accumulated extended distance (AED): The sum of distances from the mother plant cluster to the farthest ramets in each extension direction.

Extended distance (ED): The average of the three longest one-direction extension distances.

Extended area (EA): EA = π • (ED/2)².

### 2.5 Measurement of photosynthetic parameters

For photosynthetic measurements, within each block, three healthy, fully expanded, pest-free leaves with consistent light exposure were randomly selected from each germplasm (nine leaves per germplasm in total). Net photosynthetic rate (Pn), stomatal conductance (Gs), transpiration rate (Tr), and intercellular CO_2_ concentration (Ci) were measured using a portable photosynthesis system (CIRAS-4, USA). Each leaf was measured three times, and the mean value was recorded. Water-use efficiency (WUE) was calculated as: WUE = Pn/Tr.

Additionally, three sun-exposed, healthy, and fully expanded leaves at the same positional node were randomly selected from each germplasm within each block (nine leaves per germplasm) for chlorophyll content determination. Measurements were conducted in the morning on clear days in August using a handheld chlorophyll meter (SPAD-502, Konica Minolta, Japan). During measurement, the sensor clip was positioned to fully cover both optical windows of the leaf, and SPAD values were recorded after 1–2 s of stabilization. Chlorophyll content was subsequently calculated based on the corresponding calibration equation.

### 2.6 Measurement of nutrient contents

For biochemical and nutrient analyses, three individual plants were sampled from each germplasm within each block (nine plants per germplasm in total). Rhizome nodes, rhizome internodes, stems, and leaves were collected separately for subsequent measurements.

Soluble sugar content was measured using the anthrone–sulfuric acid method (Lopes et al., 2024).

Sucrose content was determined using the resorcinol colorimetric method (Teng et al., 2017).

Starch content was measured using the anthrone colorimetric method (Bahdanovich et al., 2022).

Carbon (C), nitrogen (N), phosphorus (P), and potassium (K) contents were determined following the procedure described by Zhuang and Wang (2022). Samples were ground and passed through a 60-mesh sieve, and the following methods were applied: Organic carbon (C): potassium dichromate volumetric method; Total nitrogen (N): Kjeldahl method; Total phosphorus (P): molybdenum–antimony anti-colorimetric method; Total potassium (K): flame photometry.

### 2.7 Metabolomic analysis

To investigate the metabolic basis underlying the differences in rhizome clonal propagation between the two *Leymus chinensis* germplasms, metabolomic analyses were conducted on rhizome nodes and internodes of Lc19 and Lc13, with three biological replicates per germplasm. For each biological replicate, metabolite extraction was performed with six repeated mixing cycles to ensure sufficient extraction efficiency.

All samples were freeze-dried using a vacuum lyophilizer (Scientz-100F) and subsequently ground into fine powder using a mixer mill (MM 400, Retsch) at 30 Hz for 1.5 min. Approximately 50 mg of the powdered sample was accurately weighed and extracted with 1200 μL of pre-cooled (-20 °C) 70% methanolic aqueous solution containing internal standards. The mixtures were vortexed for 30 s every 30 min for a total of six times, followed by centrifugation at 12,000 rpm for 3 min. The resulting supernatants were collected, filtered through a 0.22 μm microporous membrane, and transferred into injection vials for subsequent UPLC–MS/MS analysis.

The sample extracts were analyzed using an ultra-performance liquid chromatography–electrospray ionization–tandem mass spectrometry (UPLC–ESI–MS/MS) system, consisting of an ExionLC™ AD UPLC system coupled to a triple quadrupole–linear ion trap mass spectrometer (QTRAP, SCIEX).

Chromatographic separation was performed on an Agilent SB-C18 column (1.8 μm, 2.1 mm × 100 mm). The mobile phase consisted of solvent A (water containing 0.1% formic acid) and solvent B (acetonitrile containing 0.1% formic acid). The gradient elution program was as follows: 95% A and 5% B at the initial stage; a linear gradient to 5% A and 95% B over 9 min; holding at 5% A and 95% B for 1 min; followed by a return to 95% A and 5% B within 1.1 min and equilibration for 2.9 min. The flow rate was set at 0.35 mL min^-^¹, the column oven temperature was maintained at 40 °C, and the injection volume was 2 μL.

Mass spectrometric detection was carried out using an ESI source operating in both positive and negative ion modes. The source temperature was set to 500 °C, and the ion spray voltage was set at 5500 V in positive mode and −4500 V in negative mode. Ion source gas I (GS1), gas II (GS2), and curtain gas (CUR) were set to 50, 60, and 25 psi, respectively. Collision-activated dissociation (CAD) was set to high. Data acquisition was performed in multiple reaction monitoring (MRM) mode, with nitrogen used as the collision gas at a medium setting. Declustering potential (DP) and collision energy (CE) for individual MRM transitions were optimized to ensure optimal signal intensity. A specific set of MRM transitions was monitored according to the retention time of each metabolite.

The ESI source operation parameters were as follows: source temperature 500 °C; ion spray voltage (IS) 5500 V (positive ion mode)/-4500 V (negative ion mode); ion source gas I (GSI), gas II (GSII), curtain gas (CUR) were set at 50, 60, and 25 psi, respectively; the collision-activated dissociation (CAD) was high. QQQ scans were acquired as MRM experiments with collision gas (nitrogen) set to medium. DP (declustering potential) and CE (collision energy) for individual MRM transitions was done with further DP and CE optimization. A specific set of MRM transitions were monitored for each period according to the metabolites eluted within this period.

### 2.8 Data processing and statistical analysis

SPSS Statistics 23.0 (IBM, Armonk, NY) was used for one way analysis of variance (ANOVA) and significance test of difference. Duncan’s new multiple range test was then employed for multiple comparisons of significance of difference (*P* < 0.05), after which Origin 2021 (Origin Lab Corporation, Northampton, MA) was used for plotting. All the above data are presented in the form of mean ± SD of three replicate samples.

Correlation analysis of growth and physiological traits among different *Leymus chinensis* germplasms was performed using Origin 2021 (OriginLab Corporation, Northampton, MA, USA). Correlation analysis between metabolite abundances and phenotypic traits was conducted based on Pearson’s correlation coefficients. Corresponding p-values were calculated using two-tailed tests. To account for multiple comparisons, p-values were adjusted using the Benjamini–Hochberg false discovery rate (FDR) correction. Correlations with an adjusted p-value (FDR) ≤ 0.05 were considered statistically significant.

For metabolomic data analysis, principal component analysis (PCA) was performed using the prcomp function in R (www.r-project.org) to assess overall metabolic differences among samples. Prior to PCA, metabolite data were unit variance scaled.

Heatmap visualization was performed using the R package ComplexHeatmap. Metabolite signal intensities were Z-score normalized prior to visualization. To enhance the clarity of metabolic patterns and reduce within-group variability, biological replicates were averaged for each germplasm and tissue type before heatmap construction. Metabolites were organized according to their biochemical classes, and the heatmap was used to visualize relative abundance patterns between germplasms without hierarchical clustering.

For pairwise comparisons between germplasms, differential metabolites were identified based on variable importance in projection (VIP > 1) obtained from orthogonal partial least squares–discriminant analysis (OPLS-DA) and absolute log_2_ fold change (|log_2_FC| ≥ 1.0). OPLS-DA was conducted using the R package MetaboAnalystR, with data log_2_-transformed and mean-centered prior to analysis. To assess model robustness and avoid overfitting, permutation tests with 200 iterations were performed.

Identified metabolites were annotated using the KEGG Compound database, and annotated metabolites were subsequently mapped to metabolic pathways using the KEGG Pathway database.

## 3 Results

### 3.1 Differences in growth characteristics among *Leymus chinensis* germplasms

**Figure 2:**
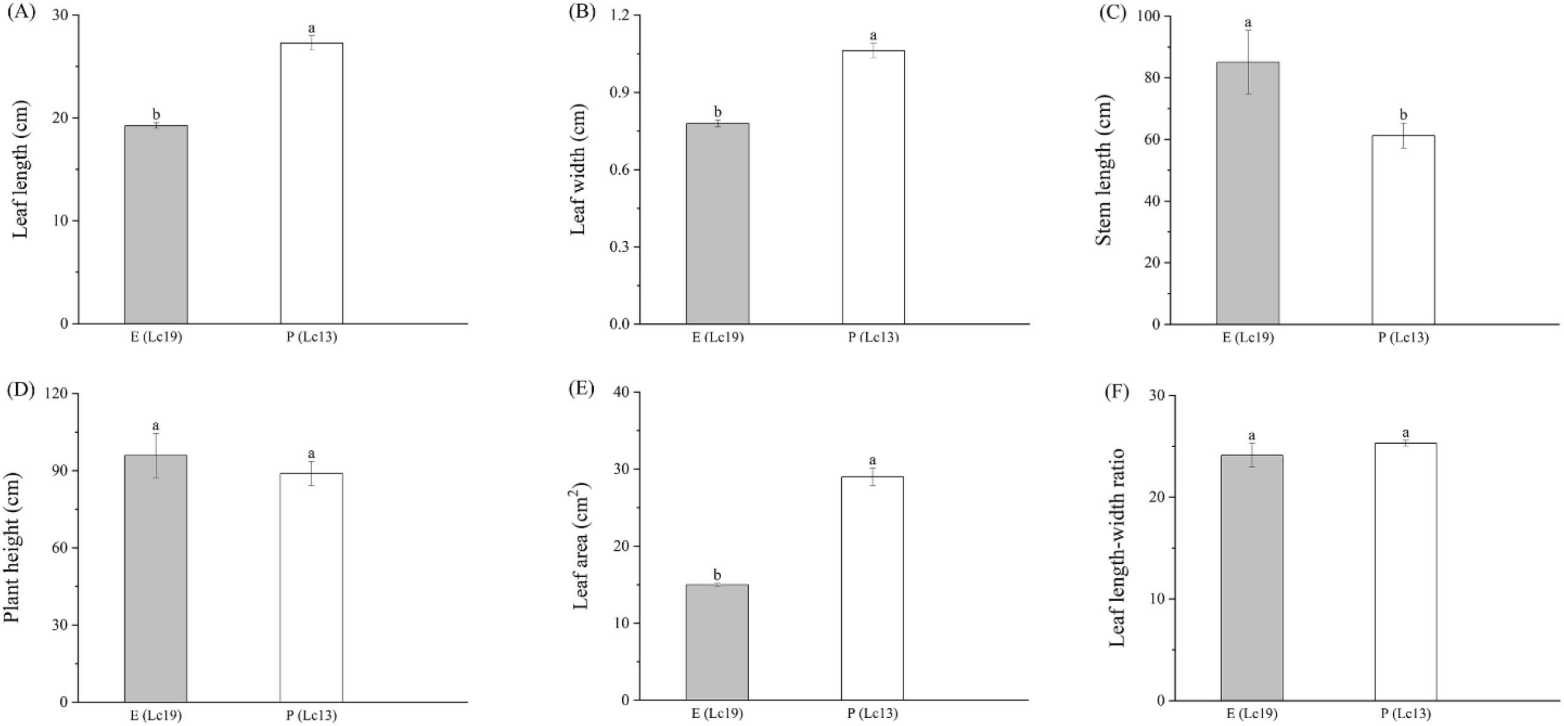
Differences in growth traits between *Leymus chinensis* germplasms with distinct rhizome propagation capacities, including **(A)** leaf length, **(B)** leaf width, **(C)** stem length, **(D)** plant height, **(E)** leaf area, and **(F)** leaf length–width ratio. E (Lc19) represents the excellent rhizome propagation germplasm, and P (Lc13) represents the poor rhizome propagation germplasm. Different lowercase letters indicate significant differences at *P* < 0.05.

Significant differences in aboveground growth were observed among *Leymus chinensis* germplasms with varying rhizome propagation capacities (**Figure 2**). Compared with P (Lc13), E (Lc19) exhibited significantly reduced leaf length, leaf width, and leaf area by 29.41%, 26.42%, and 48.27%, respectively (*P* < 0.05), whereas stem length increased significantly by 28.03% (*P* < 0.05).

**Figure 3:**
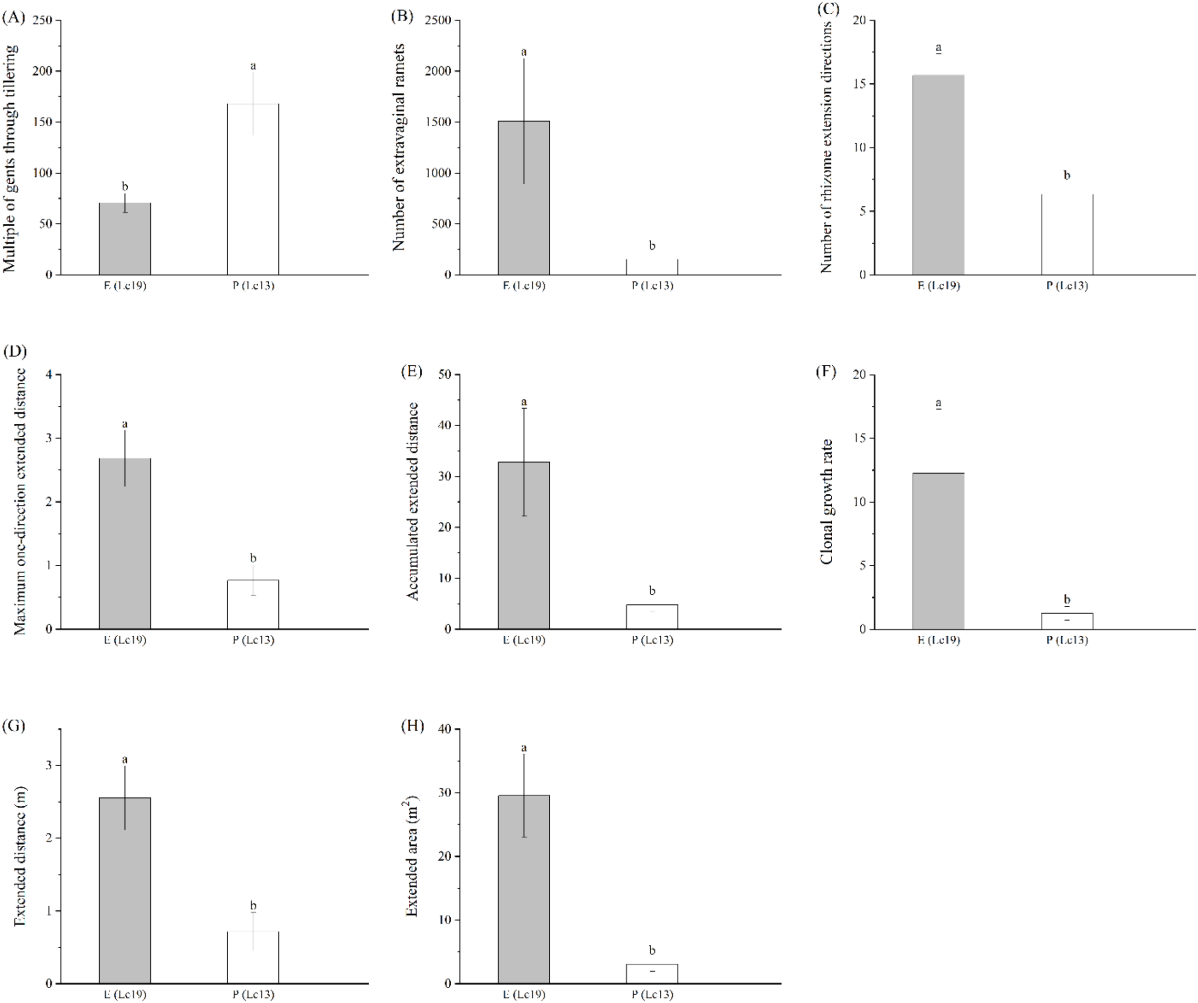
Differences in rhizome propagation indices of *Leymus chinensis* germplasms with distinct rhizome propagation abilities, including **(A)** MT, Multiple of gents through tillering, **(B)** NER, Number of extravaginal ramets, **(C)** NED, Number of rhizome extension directions, **(D)** MED, Maximum one-direction extended distance, **(E)** AED, Accumulated extended distance, **(F)** CGR, Clonal growth rate, **(G)** ED, Extended distance, and **(H)** EA, Extended area. E (Lc19) represents the excellent rhizome propagation germplasm, and P (Lc13) represents the poor rhizome propagation germplasm. Different lowercase letters indicate significant differences at *P* < 0.05.

The two *Leymus chinensis* germplasms also displayed significant differences in rhizome reproductive traits (**Figure 3**). The MT of E (Lc19) was significantly lower than that of P (Lc13) by 57.94% (*P* < 0.05), whereas all other rhizome propagation indices were significantly higher in E (Lc19) than in P (Lc13) by 71.27%, 59.60%, 89.84%, 89.81%, 71.76%, 89.71%, and 85.38%, respectively (*P* < 0.05). These results indicate that the strong clonal propagation capacity of E (Lc19) is not achieved through maternal tillering but instead relies on the rhizome system’s superior lateral extension and high efficiency in producing daughter ramets.

### 3.2 Differences in photosynthetic characteristics among *Leymus chinensis* germplasms

**Figure 4:**
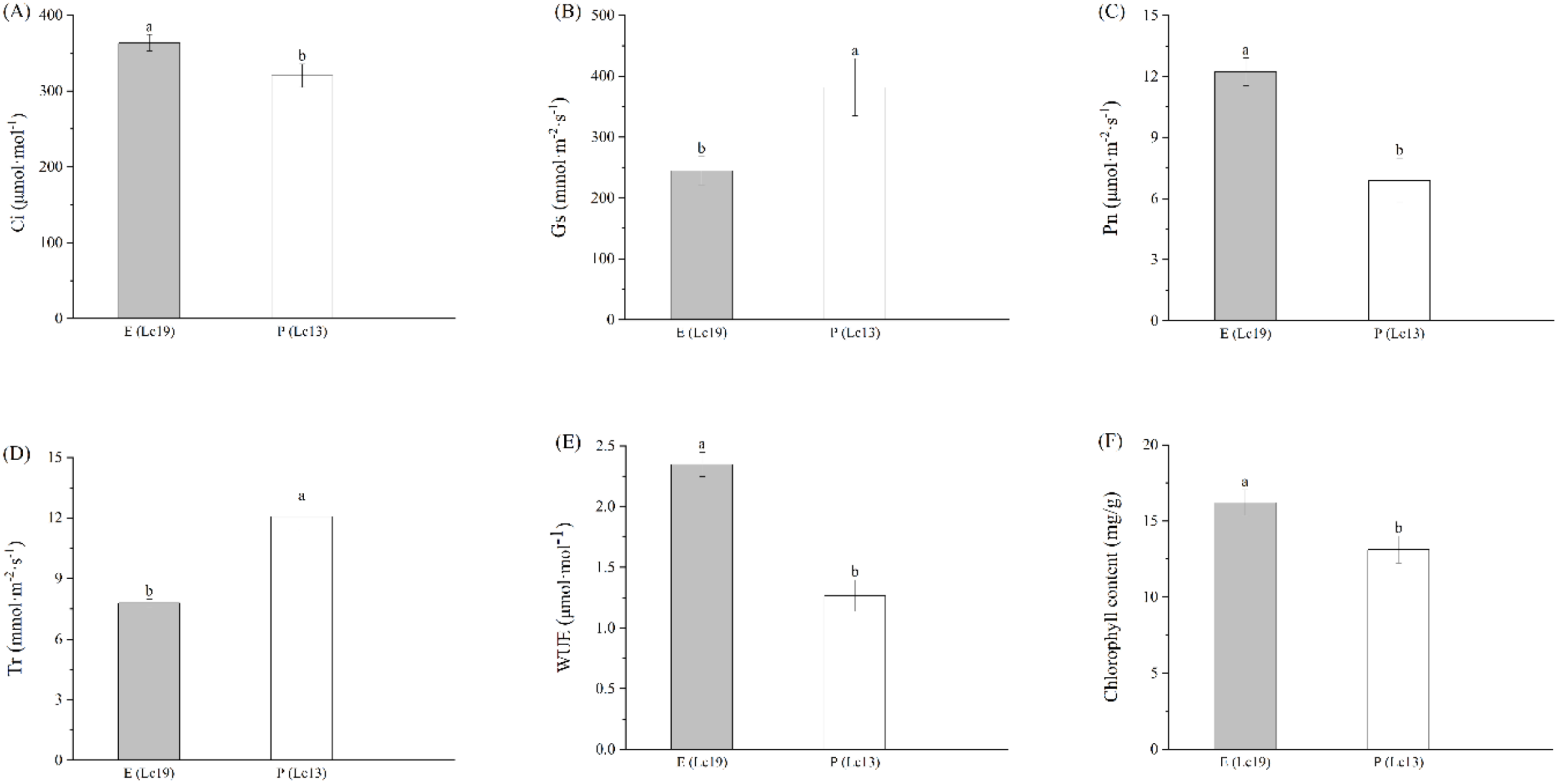
Differences in photosynthetic characteristics of *Leymus chinensis* germplasms with different rhizome propagation capacities, including **(A)** Ci, Intercellular CO_2_ concentration, **(B)** Gs, Stomatal conductance, **(C)** Pn, Net photosynthetic rate, **(D)** Tr, Transpiration rate, **(E)** WUE, Water use efficiency, and **(F)** chlorophyll content. E (Lc19) represents the excellent rhizome propagation germplasm, and P (Lc13) represents the poor rhizome propagation germplasm. Different lowercase letters indicate significant differences at *P* < 0.05.

Germplasms with different rhizome propagation capacities exhibited distinct photosynthetic strategies (**Figure 4**). Compared with P (Lc13), E (Lc19) exhibited significantly lower Gs and Tr, which were reduced by 35.87% and 35.46%, respectively. In contrast, the intercellular CO_2_ concentration (Ci), net photosynthetic rate (Pn), water-use efficiency (WUE), and chlorophyll content of E (Lc19) were significantly higher than those of P (Lc13), with increases of 11.88%, 43.70%, 45.96%, and 19.01%, respectively.

### 3.3 Differences in the allocation of nutritional substances and elements among *Leymus chinensis* germplasms

**Figure 5:**
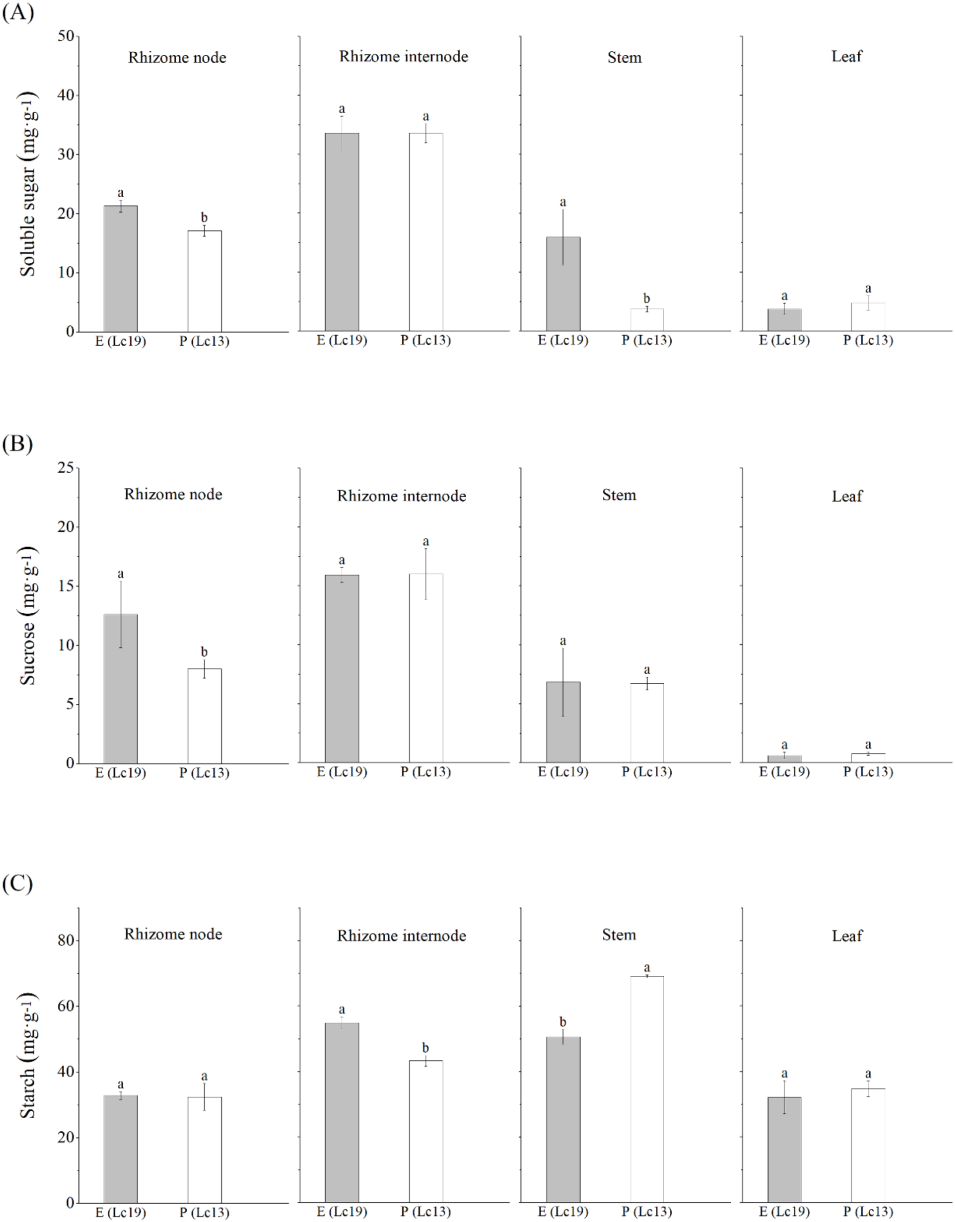
Differences in nutrient contents in different plant organs of *Leymus chinensis* with distinct rhizome propagation capacities, including **(A)** soluble sugar content, **(B)** sucrose content, and **(C)** starch content. E (Lc19) represents the excellent rhizome propagation germplasm, and P (Lc13) represents the poor rhizome propagation germplasm. Different lowercase letters indicate significant differences at *P* < 0.05.

Germplasms of *Leymus chinensis* with different rhizome propagation capacities exhibited distinct patterns of nutrient allocation (**Figure 5**). The two germplasms shared similar allocation strategies for soluble sugars and sucrose, both following the pattern: rhizome internodes > rhizome nodes > stems > leaves. This common distribution pattern indicates that rhizome internodes act as the primary “sink” organs for photosynthates in both germplasms. In contrast, starch allocation differed markedly: E (Lc19) accumulated starch mainly in rhizome internodes, followed by stems, whereas P (Lc13) accumulated starch primarily in stems, followed by rhizome internodes.

**Figure 6:**
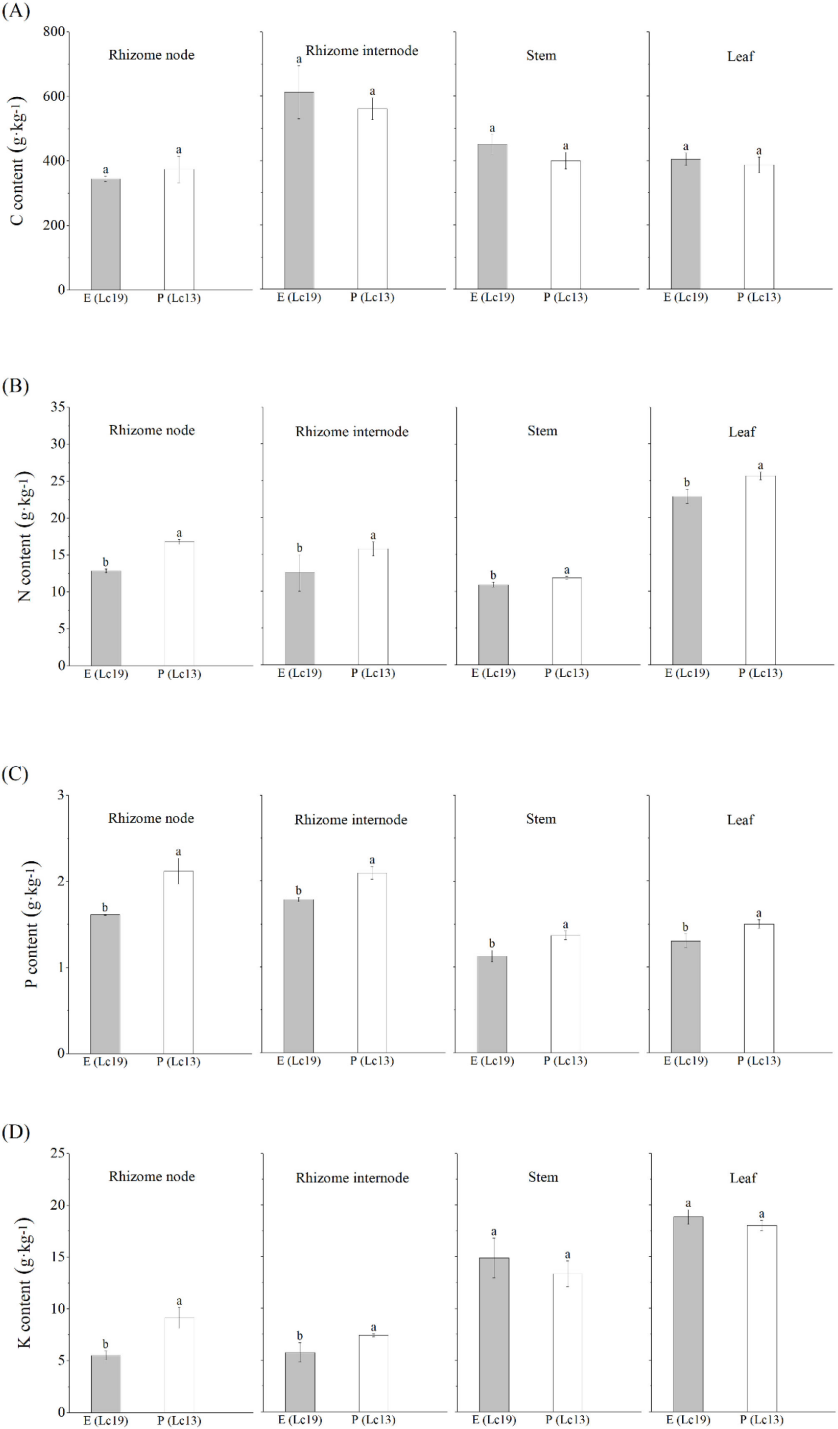
Differences in nutrient element contents in different plant organs of *Leymus chinensis* with distinct rhizome propagation capacities, including **(A)** Carbon content, **(B)** Nitrogen content, **(C)** Phosphorus content, and **(D)** Potassium content. E (Lc19) represents the excellent rhizome propagation germplasm, and P (Lc13) represents the poor rhizome propagation germplasm. Different lowercase letters indicate significant differences at *P* < 0.05.

Germplasms with different rhizome propagation capacities also showed significant differences in the distribution of nutritional elements among organs (**Figure 6**). C was enriched in rhizome internodes of both E (Lc19) and P (Lc13), with higher levels in E (Lc19). N and K were both enriched in the leaves of the two germplasms. The leaf N content of E (Lc19) was significantly lower than that of P (Lc13) by 10.82%, whereas K content was higher, consistent with the leaf’s role as the center of photosynthesis and metabolism. P was enriched in both rhizome nodes and internodes of the two germplasms, but its concentration in rhizome internodes of E (Lc19) was significantly lower than that of P (Lc13) by 14.79%.

### 3.4 Correlation analysis of growth and physiological traits in different *Leymus chinensis* germplasms

**Figure 7:**
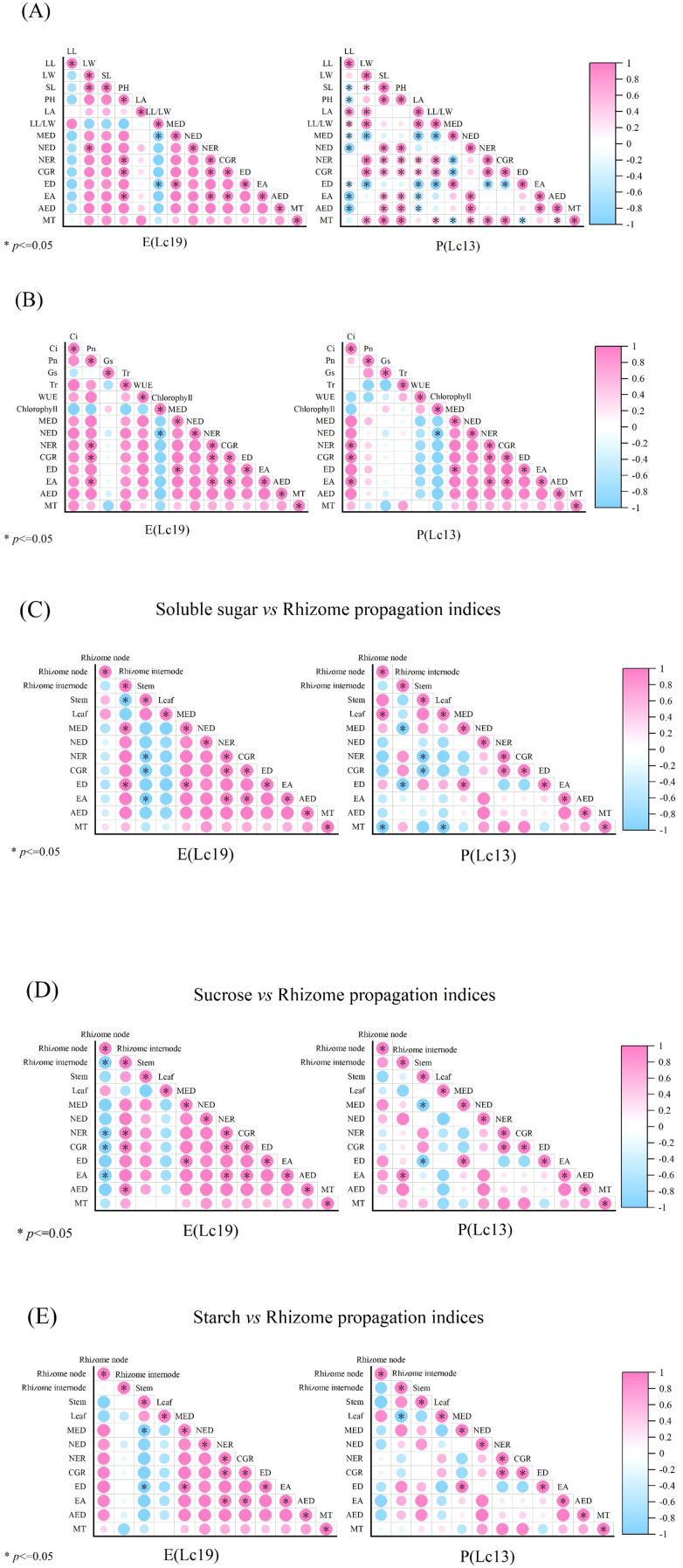
Correlation analysis between growth traits, photosynthetic characteristics, nutrient contents, and rhizome propagation indices of Leymus chinensis germplasms with contrasting rhizome propagation capacities. **(A)** Correlation analysis between aboveground growth traits and rhizome propagation indices; **(B)** correlation analysis between photosynthetic characteristics and rhizome propagation indices; **(C)** correlation analysis between soluble sugar content and rhizome propagation indices; **(D)** correlation analysis between sucrose content and rhizome propagation indices; **(E)** correlation analysis between starch content and rhizome propagation indices.

Correlation analyses among growth traits, photosynthetic characteristics, nutritional substances, and rhizome reproductive indices of the two germplasms (Lc19 and Lc13) revealed pronounced differences in their growth and propagation strategies (**Figure 7**). In E (Lc19), rhizome reproductive indices were significantly positively correlated with leaf width, stem length, and plant height, but negatively correlated with leaf length and leaf length–width ratio. Regarding photosynthetic traits, rhizome reproductive indices were significantly positively correlated with Ci, Pn, Tr, and WUE. In terms of nutritional substances, starch content in rhizome internodes was significantly positively correlated with CGR, whereas soluble sugars in stems and sucrose in rhizomes showed negative correlations with several rhizome reproductive indices. In contrast, reproductive indices of P (Lc13) were associated with aboveground growth, with MT, NER, and CGR all showing significant positive correlations with leaf width, stem length, and plant height. For photosynthetic traits, rhizome reproductive indices were significantly positively correlated only with Ci. In terms of nutritional substances, sucrose content in rhizome internodes was significantly positively correlated with EA, whereas soluble sugars in rhizome nodes and sucrose in stems were significantly negatively correlated with MED and ED.

### 3.5 Metabolomic analysis of different *Leymus chinensis* germplasms

**Figure 8:**
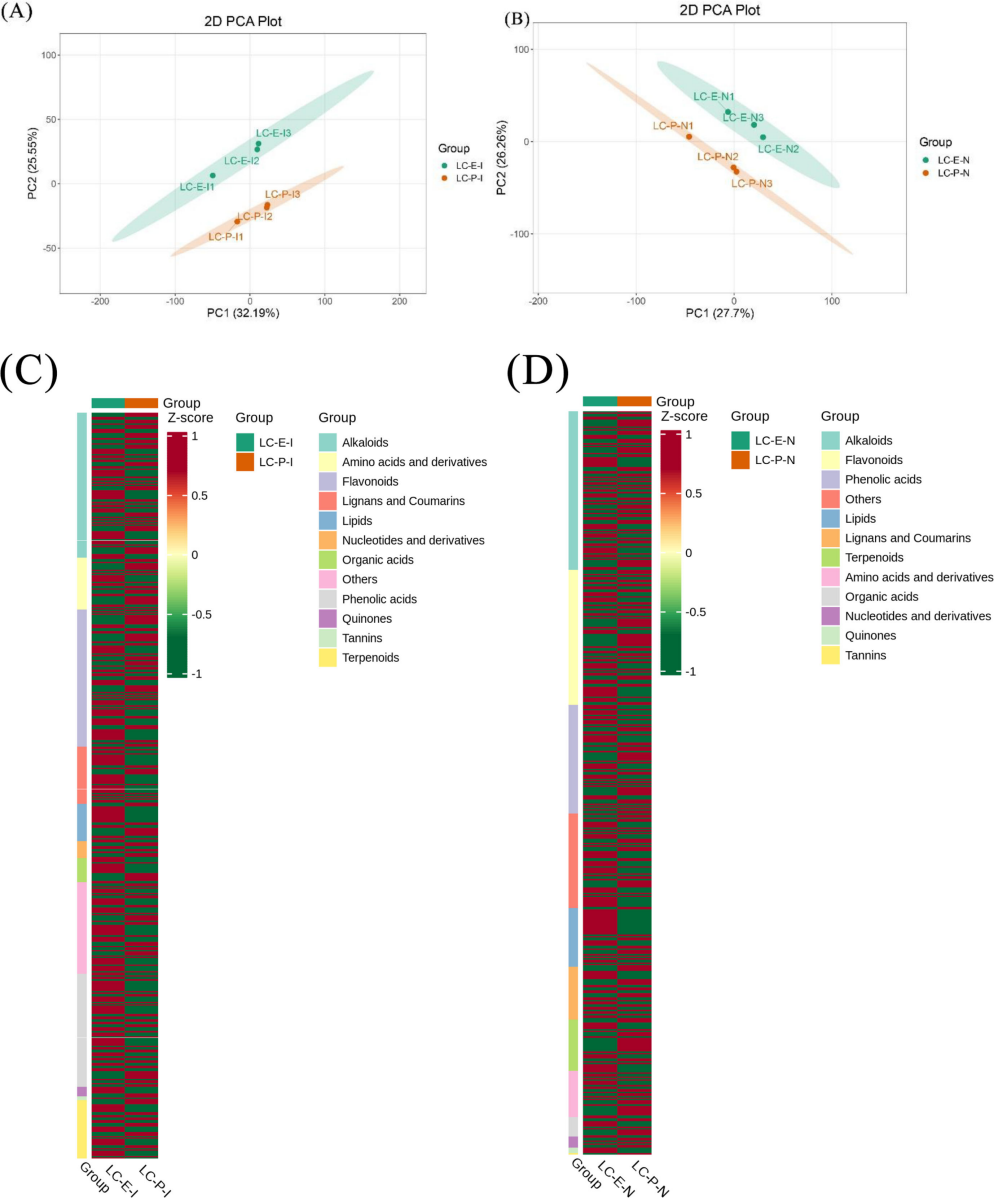
PCA plots and metabolite class–organized heatmaps of differential metabolites in rhizome internodes and rhizome nodes of *Leymus chinensis* germplasms with contrasting rhizome propagation capacities. **(A)** PCA plot of E-I vs. P-I; **(B)** PCA plot of E-N vs. P-N; **(C)** heatmap of differential metabolites in rhizome internodes (E-I vs P-I) organized by metabolite classes; **(D)** heatmap of differential metabolites in rhizome nodes (E-N vs P-N) organized by metabolite classes. For the heatmaps **(C, D)**, metabolite abundances were Z-score normalized, and biological replicates were averaged for each germplasm prior to visualization. Metabolites were arranged according to their biochemical classes to facilitate comparison of differential accumulation patterns between germplasms.

To elucidate the metabolic basis underlying the differences in rhizome propagation capacity between the two *Leymus chinensis* germplasms, a non-targeted metabolomic analysis was performed on rhizome nodes and internodes. Principal component analysis (PCA) showed a clear separation between E (Lc19) and P (Lc13) in the metabolic space (**Figures 8A, B**), indicating pronounced differences in their overall metabolic profiles. This separation was primarily associated with coordinated shifts in multiple metabolite classes that differed significantly between the two germplasms. Heatmap visualization based on group-averaged metabolite profiles further illustrated distinct metabolite accumulation patterns between E and P (**Figures 8C, D**). Metabolites were organized according to their biochemical classes to facilitate interpretation of differential trends. Amino acids and their derivatives, lipids, organic acids, and several flavonoids and phenolic acids were generally enriched in E-I and E-N, whereas nucleosides and their derivatives, tannins, and quinones showed higher relative abundances in P-I and P-N. These metabolite categories therefore represent the main contributors to the metabolic differentiation observed in the PCA.

**Figure 9:**
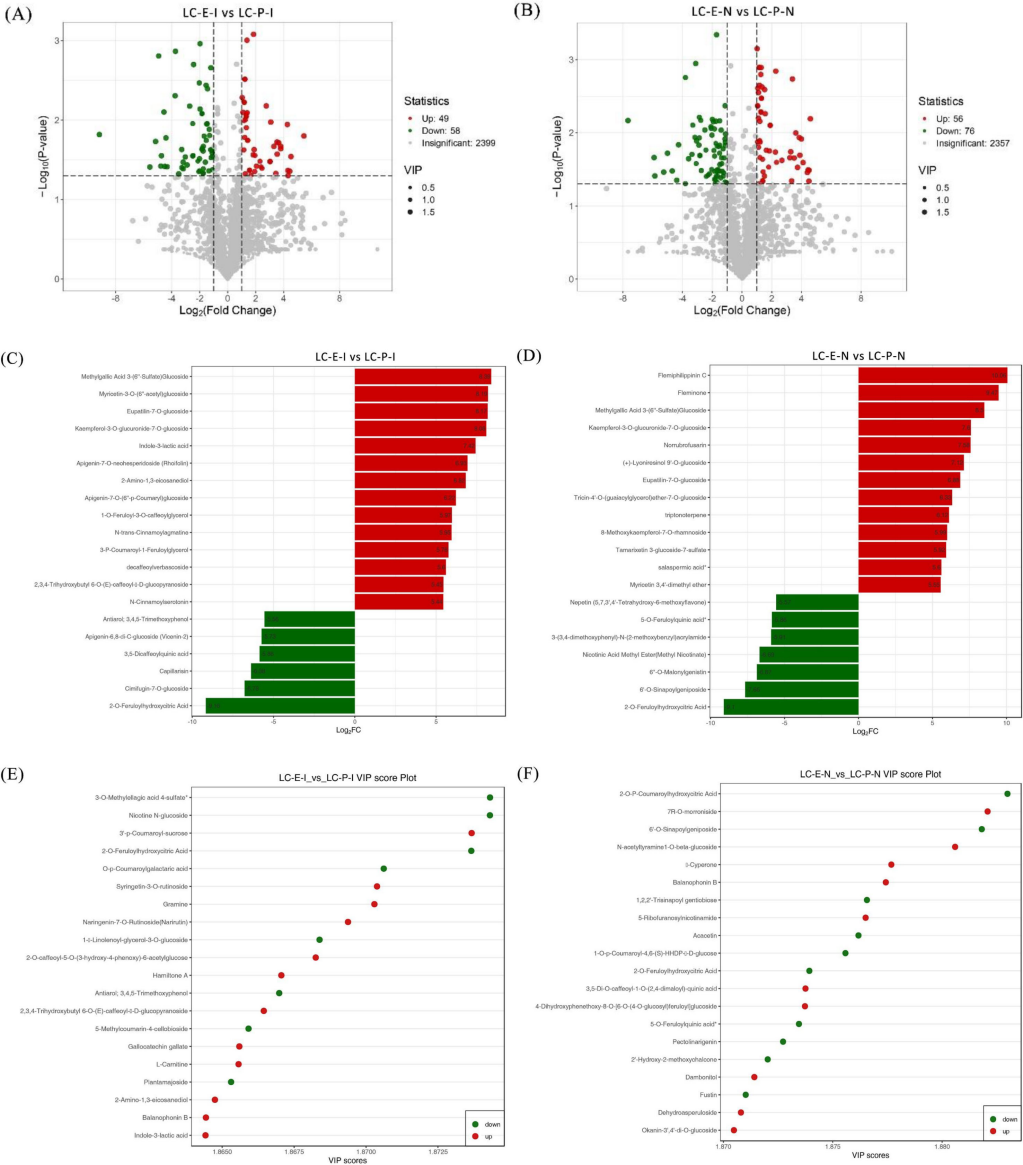
Volcano plots, fold-change bar charts, and VIP value plots of differential metabolites in rhizome internodes and rhizome nodes of *Leymus chinensis* germplasms with different rhizome propagation capacities: **(A)** volcano plot of E-I vs. P-I; **(B)** volcano plot of E-N vs. P-N; **(C)** fold-change plot of E-I vs. P-I; **(D)** fold-change plot of E-N vs. P-N; **(E)** VIP plot of E-I vs. P-I; and **(F)** VIP plot of E-N vs. P-N.

In the comparison of E-I vs. P-I, 107 significantly differential metabolites were identified, including 49 upregulated and 58 downregulated metabolites (**Figure 9A**). In the E-N vs. P-N comparison, 132 significantly differential metabolites were identified, with 56 upregulated and 76 downregulated (**Figure 9B**). To identify key metabolites contributing most to the observed differences, fold-change bar plots and VIP (variable importance in projection) values were jointly analyzed (**Figures 9C–F**). The results showed that in E-I, metabolites such as 2,3,4-Trihydroxybutyl 6-O-(E)-caffeoyl-β-D-glucopyranoside, indole-3-lactic acid, and 2-amino-1,3-eicosanediol were significantly upregulated with high VIP values. In E-N, flemiphilippinin C, fleminone, and methylgallic acid 3-(6”-sulfate) glucoside were significantly upregulated. In contrast, in P-I, phenolic acids such as 2-O-feruloylhydroxycitric acid and antiarol;3,4,5-trimethoxyphenol were significantly upregulated with high VIP values. In P-N, metabolites including 6’-O-sinapoylgeniposide, 2-O-feruloylhydroxycitric acid, and 5-O-feruloylquinic acid* were markedly upregulated with high VIP values. These findings indicate pronounced metabolic differentiation between the two germplasms.

**Figure 10:**
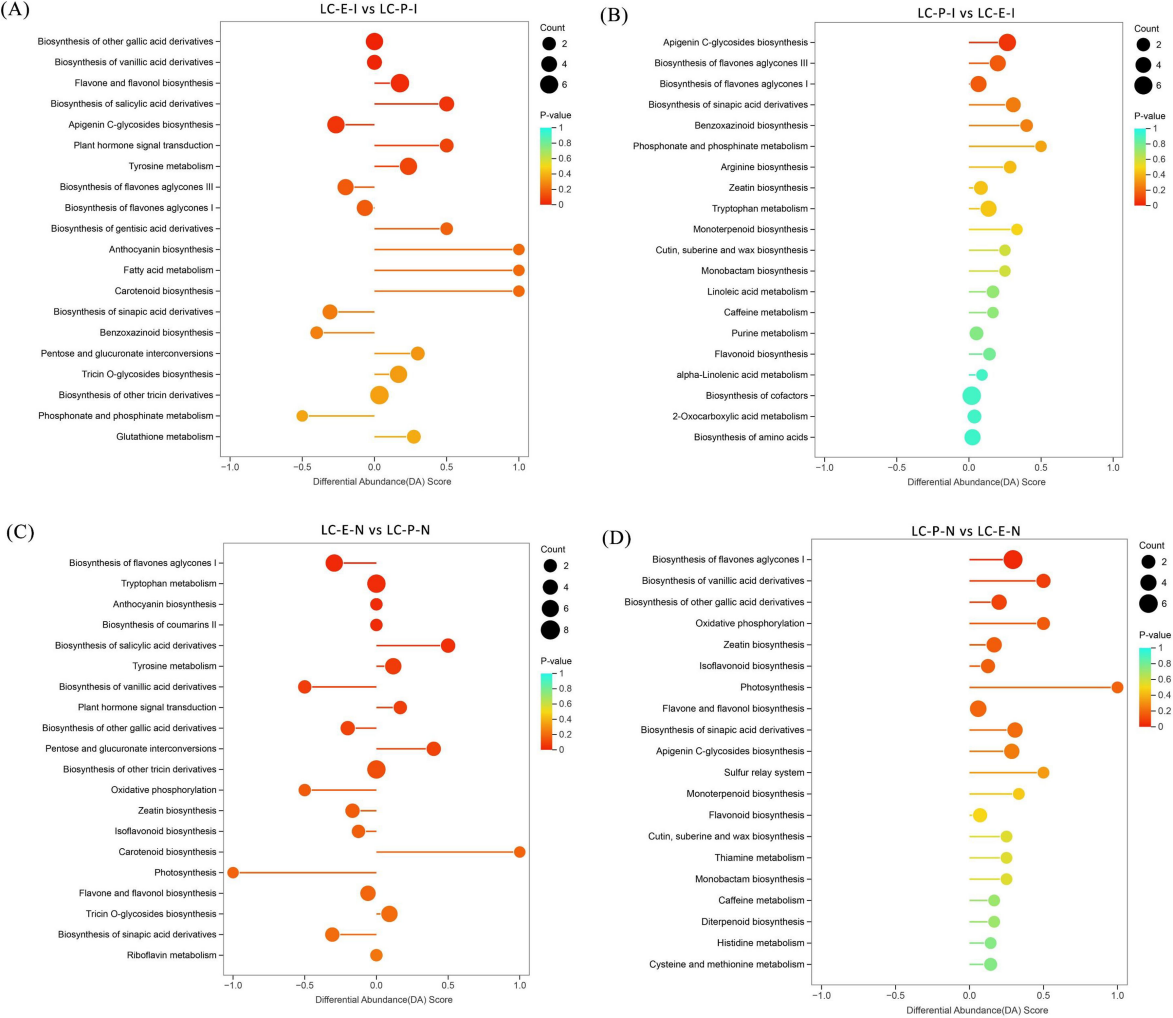
Differential abundance score plots of rhizome internodes and rhizome nodes of *Leymus chinensis* germplasms with distinct rhizome propagation capacities: **(A)** differential abundance score plot of E-I vs. P-I; **(B)** differential abundance score plot of P-I vs. E-I; **(C)** differential abundance score plot of E-N vs. P-N; and **(D)** differential abundance score plot of P-N vs. E-N.

To elucidate the biological significance of the coordinated action of these differential metabolites, KEGG pathway enrichment analysis was conducted. As shown in **Figure 10**, differential metabolites in E-I were significantly enriched in pathways such as fatty acid metabolism and carotenoid biosynthesis. In E-N, differential metabolites were significantly enriched in growth-promoting pathways, including biosynthesis of flavone aglycones I, tryptophan metabolism, and anthocyanin biosynthesis. In P-I, differential metabolites were mainly enriched in apigenin C-glycoside biosynthesis, biosynthesis of flavone aglycones III, and biosynthesis of flavone aglycones I. In P-N, differential metabolites were enriched primarily in defense-related pathways such as biosynthesis of flavone aglycones I, biosynthesis of vanillic acid derivatives, and biosynthesis of other gallic acid derivatives. These findings demonstrate fundamental functional differentiation in the metabolic regulatory networks of rhizome internodes and nodes between the two germplasms, providing key metabolic evidence for understanding their different rhizome propagation capacities.

To facilitate an integrated interpretation of the physiological and metabolic differences between the two germplasms, a summary table was constructed to link the contrasting physiological strategies with their representative differential metabolites and associated metabolic pathways (**Table 2**. The comprehensive metabolite dataset and the detailed KEGG pathway enrichment analysis are presented in **Supplementary Table 1** and **Supplementary Table 2**, respectively).

**Table 2:** Summary of physiological strategies, representative germplasms, key differential metabolites, and dominant metabolic pathways identified in *Leymus chinensis* germplasms with contrasting rhizome propagation capacities.

Physiological strategy	Representative germplasm	Representative metabolites (functional groups)	Dominant metabolic pathways
Belowground-investment strategy (rhizome expansion–oriented)	E (Lc19)	Auxin-related metabolite: Indole-3-lactic acid Phenolic acid derivatives: 2,3,4-Trihydroxybutyl 6-O-(E)-caffeoyl-β-D-glucopyranoside; Methylgallic acid 3-(6″-sulfate) glucoside Lipid-related metabolite: 2-Amino-1,3-eicosanediol Flavonoids: Flemiphilippinin C; Fleminone	Biosynthesis of flavones aglycones l Tryptophan metabolism Anthocyanin biosynthesis Fatty acid metabolism Carotenoid biosynthesis
Aboveground-maintenance strategy (physiological stability–oriented)	P (Lc13)	Phenolic acid derivatives: 2-O-Feruloylhydroxycitric acid; 5-O-Feruloylquinic acid Phenolic compounds: Antiarol; 3,4,5-Trimethoxyphenol Iridoid glycoside: 6′-O-Sinapoylgeniposide	Apigenin C-glycosides biosynthesis Biosynthesis of flavones aglycones l Biosynthesis of vanillic acid derivatives Biosynthesis of other gallic acid derivatives

E (Lc19) represents the germplasm with strong rhizome propagation capacity, whereas P (Lc13) represents the germplasm with weak rhizome propagation capacity.

## 4 Discussion

Clonal propagation through rhizomes was central to the population renewal and ecological adaptation of *Leymus chinensis*. By conducting an integrated analysis of two *Leymus chinensis* germplasms differing in rhizome propagation capacity, this study not only elucidated the physiological basis underlying their phenotypic differentiation but also, for the first time, systematically clarified the regulatory networks at the metabolic level. The results indicated that the differences between E (Lc19) and P (Lc13) originated from fundamentally distinct source–sink relationships and resource allocation strategies, which in turn drove their different metabolic processes.

### 4.1 Analysis of growth and physiological characteristics of *Leymus chinensis* germplasms with differing rhizome propagation capacities

This study demonstrated that E (Lc19) and P (Lc13) exhibited markedly divergent physiological strategies, which directly determined their different rhizome propagation capacities. E (Lc19) exhibited a typical “belowground-investment” strategy, characterized by a morphological pattern of “small leaves and elongated stems,” reflecting optimized resource allocation in which a larger proportion of assimilates was transported from the leaves to belowground tissues through stem elongation. The stem thus served as a conduit between the mother plant and distant clonal ramets. Such a relationship between rhizome propagation capability and lateral resource allocation had been documented in studies of perennial clonal plants (Caplan et al., 2014; Jitka et al., 2018). This observation was consistent with the rhizome reproduction indices measured in this study: except for MT, all lateral propagation indices (e.g., NER, NED, MED) were significantly higher in E (Lc19) than in P (Lc13), indicating that its propagation depended on the spatial propagation of the rhizome system rather than on localized tillering around the mother plant. Notably, E (Lc19) maintained relatively low Gs and Tr while exhibiting higher Pn, WUE, and Ci, indicating that its belowground sink demand was met through enhanced resource-use efficiency per unit input. This “conservative yet efficient” photosynthetic strategy aligned with mechanisms by which clonal plants sustained propagation under resource-limited conditions through efficient resource utilization (Jana and Jan, 2024). Meanwhile, E (Lc19) exhibited a pronounced belowground bias in nutrient allocation, with starch predominantly accumulating in rhizome internodes, thereby providing a direct energy source for rhizome propagation. Starch content in rhizome internodes was also significantly positively correlated with CGR. In contrast, P (Lc13) followed an “aboveground-maintenance” strategy. Its larger leaf area and higher Gs and Tr indicated a growth strategy that relied on gas exchange and aboveground biomass accumulation to compete for light resources. Its rhizome system functioned primarily in storage and maintenance; although maternal tillering capacity was relatively strong, its lateral propagation capacity was weak, suggesting that its propagation was biased toward consolidating growth around the mother plant. These different strategies reflected two adaptive approaches evolved by clonal plants to cope with heterogeneous environments: one prioritizing propagation and the other emphasizing consolidation (Yu et al., 2020; Wang et al., 2020b).

### 4.2 Analysis of metabolic differences between *Leymus chinensis* germplasms with distinct rhizome propagation capacities

Metabolomic analysis provided direct system-level evidence supporting the differentiation of the above physiological strategies, demonstrating that the two germplasms possessed functionally distinct metabolic networks within their rhizome internodes. The rhizome nodes and internodes of E (Lc19), functioning as the most metabolically active sites of belowground propagation, were characterized by substantial accumulation of amino acids, organic acids, and lipids—key structural units that provided essential precursors and bioenergetic support for active protein synthesis, cell division, and elongation (Trovato et al., 2021). At the metabolite level, compounds such as indole-3-lactic acid and 2-amino-1,3-eicosanediol were significantly enriched in E (Lc19), indicating enhanced hormonal regulation and membrane lipid remodeling, both of which were critical for sustained rhizome elongation and tissue expansion (Qiu et al., 2025). Meanwhile, metabolic pathways such as biosynthesis of flavones aglycones I, tryptophan metabolism, and anthocyanin biosynthesis were broadly upregulated in E-N. These pathways were supported by the accumulation of specific phenolic and indole-derived metabolites, including 2,3,4-trihydroxybutyl 6-O-(E)-caffeoyl-β-D-glucopyranoside and flemiphilippinin C, which were known to possess strong antioxidant capacity and to participate in growth-related secondary metabolism. Tryptophan metabolism provided key developmental momentum for rapid rhizome elongation, whereas flavone aglycones and anthocyanins, through their strong antioxidant activities, offered essential cellular protection during rapid growth and helped maintain intracellular homeostasis (Wang et al., 2025; Daryanavard et al., 2023). Moreover, the activation of the fatty acid metabolism and carotenoid biosynthesis pathways provided the structural foundation for membrane formation, precursors for signaling molecules (such as JA and ABA), and enhanced cellular protective capacity to support rhizome elongation (Cerqueira et al., 2023; Beltran and Stange, 2016). Together, the coordinated enrichment of these metabolites and pathways was closely associated with the higher rhizome elongation rate and greater belowground biomass investment observed in E (Lc19).

In sharp contrast, the rhizome internodes of P (Lc13) resembled a “fortress of defense and maintenance.” Its metabolic profile emphasized the maintenance of core physiological homeostasis rather than structural formation. Enrichment of the apigenin C-glycosides biosynthesis pathway indicated substantial accumulation of stable C-glycosylated flavonoids, which possessed durable antioxidant and antimicrobial activities (Wang et al., 2020a). This pattern was supported by the elevated levels of metabolites such as 2-O-feruloylhydroxycitric acid, 5-O-feruloylquinic acid, and 6’-O-sinapoylgeniposide, all of which were associated with antioxidant defense and stress tolerance. Additionally, enhancement of biosynthesis of flavones aglycones reflected active synthesis and transformation of flavonoid backbones, increasing rhizome ROS-scavenging capacity and contributing to defense signal regulation (Şabik et al., 2024). Moreover, upregulation of the biosynthesis of other gallic acid derivatives pathway indicated increased accumulation of gallic acid–derived antioxidants, which helped alleviate oxidative stress and enhance tissue stability (Wang et al., 2019). These metabolite-supported pathways were consistent with the reduced rhizome extension capacity but enhanced stress resistance and physiological homeostasis observed in P (Lc13).

Thus, while the metabolic network of E (Lc19) centered on promoting morphological construction and active propagation, that of P (Lc13) focused on ensuring survival, maintaining internal homeostasis, and executing defensive functions, providing intrinsic metabolic support for its limited belowground growth capacity.

### 4.3 Ecological and restorative implications of contrasting rhizome propagation strategies in *Leymus chinensis* germplasms

Both *Leymus chinensis* germplasms originated from the Eurasian steppe and are geographically proximate, yet they exhibit pronounced divergence in rhizome propagation strategies. This differentiation reflects long-term adaptive responses to soil heterogeneity and environmental constraints, and it provides important insights for grassland restoration and management (Liu et al., 2025). Rhizomatous clonal plants do not rely on a single optimal strategy; rather, their persistence and spatial occupation depend on how physiological performance and metabolic regulation are coordinated with specific soil conditions, resource availability, and disturbance regimes.

Germplasms characterized by strong rhizome propagation capacity, such as E (Lc19), are particularly suitable for restoration scenarios where rapid spatial occupation is required. In soils with relatively sufficient moisture or patchy nutrient distribution, extensive rhizome elongation enables efficient lateral foraging, promotes rapid ground cover formation, and accelerates the stabilization of degraded surfaces. Such traits are especially advantageous during the early stages of restoration, when vegetation cover is sparse and erosion risk is high. By quickly establishing interconnected clonal networks, these germplasms can enhance soil structure, reduce surface disturbance, and facilitate subsequent community development. In contrast, germplasms with conservative rhizome propagation strategies, represented by P (Lc13), are better suited to environments characterized by chronic edaphic stress, such as saline–alkali soils, nutrient-poor substrates, or sites with long-term water limitation. Under these conditions, excessive belowground propagation may impose high metabolic costs without proportional ecological returns. A strategy emphasizing localized persistence, physiological stability, and stress tolerance can therefore confer greater long-term survival and community resilience. Such germplasms are more appropriate for maintaining vegetation stability and preventing further degradation in marginal or highly stressed habitats.

Importantly, the ecological value of these two strategies lies not in their mutual exclusivity but in their complementarity. Grassland ecosystems are inherently heterogeneous, both spatially and temporally, and restoration outcomes depend on matching plant functional traits with site-specific soil constraints and management objectives. Integrating germplasms with different rhizome propagation strategies can enhance restoration flexibility, allowing practitioners to balance rapid establishment with long-term stability across heterogeneous landscapes. From a broader ecological restoration perspective, recognizing and utilizing strategy differentiation in rhizomatous clonal plants offers a pathway toward precision restoration under future climate uncertainty. As climate change is expected to intensify soil moisture variability and increase the frequency of extreme stress events, restoration approaches based on uniform germplasm selection are likely to be insufficient. Strategy-oriented germplasm deployment, informed by soil environmental conditions and restoration goals, can improve restoration efficiency, reduce failure risks, and enhance the adaptive capacity of restored grasslands. Overall, this study highlights that rhizome propagation strategies are not merely physiological traits but functional tools that link plant adaptation, soil environments, and restoration practice. By translating intraspecific strategy differentiation into practical guidelines for germplasm utilization, this work contributes to the development of more effective, resilient, and ecologically informed grassland restoration strategies.

## 5 Conclusions

The findings of this study reveal that the differences in rhizome propagation capacity between the *Leymus chinensis* germplasms E (Lc19) and P (Lc13) arise from their fundamentally distinct ecological adaptation strategies and the corresponding physiological and metabolic regulatory mechanisms. At the physiological level, E (Lc19) exhibits a typical belowground resource allocation tendency, characterized by a “small-leaf, long-culm” morphology, higher water-use efficiency, and preferential allocation of starch and other storage substances to rhizome internodes, thereby supporting its strong clonal propagation capacity. In contrast, P (Lc13) adopts an aboveground maintenance-oriented strategy, in which its larger leaf area, higher transpiration rate, and starch accumulation pattern in stems favor aboveground photosynthetic competition and structural maintenance. At the metabolic level, the rhizome nodes and internodes of E (Lc19) display an “active expansion–oriented” metabolic profile. The upregulation of pathways such as tryptophan metabolism, biosynthesis of flavones aglycones I, and anthocyanin biosynthesis enhances growth potential and antioxidant capacity. Furthermore, the accumulation of auxin-related metabolites and the activation of fatty acid and carotenoid metabolism further support rapid rhizome construction. In contrast, the rhizome internodes and nodes of P (Lc13) exhibit a “homeostasis-maintenance–oriented” metabolic profile. The enrichment of apigenin C-glycosides biosynthesis, biosynthesis of flavones aglycones, and biosynthesis of other gallic acid derivatives strengthens sustained antioxidant and defense functions, thereby limiting metabolic investment in morphological construction. The coordinated differentiation in physiology and metabolism between the two germplasms represents adaptive outcomes shaped by long-term natural selection under heterogeneous habitats, reflecting the ecological trade-off of clonal plants between “exploration” and “conservation.” This study provides new theoretical foundations for understanding the metabolic mechanisms underlying plant ecological adaptation strategies and offers a reference framework for the differentiated utilization of germplasms with distinct propagation strategies, with direct implications for supporting grassland management and restoration planning in heterogeneous environments.

## Data Availability

The original contributions presented in the study are included in the article/**Supplementary Material**. Further inquiries can be directed to the corresponding author.
