# Correction: Differentiation mechanisms of rhizome clonal propagation strategies in *Leymus chinensis* based on physiological and metabolomic analyses

**DOI:** 10.3389/fpls.2026.1827524

**Published:** 2026-03-20

**Authors:** Yahui Zhang, Shimeng Zhao, Xiangyang Hou

**Affiliations:** 1 College of Grassland Science, Shanxi Agricultural University, Jinzhong, China; 2 Key Laboratory for Model Innovation in Forage Production Efficiency, Ministry of Agriculture and Rural Affairs, Jinzhong, China

**Keywords:** belowground–aboveground allocation, clonal plant ecology, physiological–metabolic regulation, rhizome-mediated clonal growth, source–sink relationship

An incorrect **Funding** statement was provided. The statement originally stated “The author(s) declare that financial support was received for the research and/or publication of this article. This work was supported by the Key Research and Development Projects in Shanxi Province (202102140601006).” The correct statement is “The author(s) declare that financial support was received for the research and/or publication of this article. This work was supported by the National Key Research and Development Program of China (2022YFF1302803).”

The original version of this article has been updated.

